# Orthonasal and retronasal odor identification in patients with parosmia

**DOI:** 10.1007/s00405-023-08072-z

**Published:** 2023-06-20

**Authors:** Shubin Li, Paolo Boscolo-Rizzo, Francesco Uderzo, Giancarlo Tirelli, Katherine L. Whitcroft, Thomas Hummel

**Affiliations:** 1 Department of Otorhinolaryngology, Smell and Taste Clinic, Technical University of Dresden, Fetscherstrasse. 74, 01307 Dresden, Germany; 2 Department of Medical, Surgical and Health Sciences, Section of Otolaryngology, University of Trieste, Trieste, Italy; 3 UCL Ear Institute, Faculty of Brain Sciences, University College London, London, UK

**Keywords:** Orthonasal function, Retronasal function, Parosmia, COVID-19, Olfactory dysfunction

## Abstract

**Objective:**

To compare retronasal and orthonasal perception in parosmic COVID-19 patients, in order to determine whether COVID-19 has a differential effect on these functions.

**Methods:**

Using the Sniffin Sticks test battery orthonasal function was examined for odor threshold, discrimination and identification. Retronasal function was assessed using 20 tasteless aromatized powders. Gustatory function was measured using the Taste Strips test.

**Results:**

This study included 177 patients (127 women, 50 men; mean age 45 years), of whom 127 (72%) were hyposmic and 50 (28%) normosmic. Compared to patients without parosmia, parosmic patients performed worse in odor identification for both orthonasal (*F* = 4.94, *p* = 0.03) and retronasal tests (*F* = 11.95, *p* < 0.01). However, an interaction effect between route of odor identification (orthonasal or retronasal) and parosmia status was found (*F* = 4.67, *p* = 0.03): patients with parosmia had relatively lower retronasal scores than patients without parosmia.

**Conclusion:**

Our results suggest that COVID-19 may affect the olfactory mucosa differently along the anterior–posterior axis, thereby possibly contributing to the pathophysiology of parosmia. Patients with parosmia also exhibit a higher degree of impairment when odors are presented through the retronasal route during eating and drinking.

## Introduction

Many studies have reported olfactory and gustatory dysfunction in COVID-19, including anosmia, hyposmia, ageusia and hypogeusia [1, 2]. About 80% of patients recover within 4 weeks, but some experience smell distortions during the course of their recovery [3, 4]. COVID-19-associated parosmia is an important issue because it significantly affects quality of life [5–7].

An abnormal eating experience is another important issue caused by COVID-19, which results not only from gustatory but also retronasal olfactory dysfunction [8–10]. However, in COVID-19-related research, retronasal dysfunction has received far less attention than orthonasal olfaction.

It has been suggested that retronasal olfaction is processed differently than orthonasal olfaction [11]. In patients, retronasal olfactory dysfunction is often confused with gustatory dysfunction [9]. Previous research even found that a major portion of the patients reporting altered taste perception showed normal gustatory function but olfactory impairments [12, 13]. The aim of the present study was to compare retronasal and orthonasal perception in parosmic COVID-19 patients, in order to examine whether COVID-19 has a differential effect on these functions.

## Materials and methods

### Study design and population

This study was conducted according to the guidelines of the Declaration of Helsinki and was approved by the relevant ethics committees. All patients had been referred to the ear, nose and throat outpatient clinic for taste and smell disorders, between February 2021 and September 2022. The inclusion criteria were age ≥ 18 years, onset of smell dysfunction during the acute phase of RT-PCR confirmed SARS-CoV-2 infection and persisting for more than 3 months, and psychophysical evaluation of the orthonasal and retronasal olfactory function. The exclusion criteria were history of major head trauma, history of previous sinonasal surgery, neurological/psychiatric disorders, and pre-existing olfactory/gustatory dysfunction. Written informed consent was obtained from all participants.

### Self-reported and psychophysical olfactory and gustatory assessment

Patients were asked about the presence of parosmia (“Do you smell odors differently compared to previous experiences?”) and phantosmia (“Do you smell odors in the absence of an apparent source?”) based on a binary outcome of yes and no.

Psychophysical orthonasal olfactory function was assessed using the validated extended Sniffin’ Sticks test battery (Burghart Messtechnik, Holm, Germany) including phenylethyl-alcohol (PEA) odor thresholds, odor discrimination, and odor identification [14]. Retronasal olfactory function was tested using 20 powdered tasteless aromas (Givaudan Schweiz AG, Dubendorf, Switzerland) [10]. Gustatory assessment was performed using the Taste Strips test (Taste Strips, Burghart Messtechnik, Holm, Germany) according to a standardized protocol [15]. Orthonasal function was expressed through a Threshold, Discrimination, and Identification (TDI) score, indicating normosmia (TDI ≥ 30.75), hyposmia (TDI 16.25–30.50) and anosmia (TDI ≤ 16.0) [16]. Gustatory function was measured using the Taste Strips test. A Taste Strips Score (TSS) was calculated and used for the identification of hypogeusia (TSS < 9 points) and normogeusia (TSS ≥ 9 points).

### Statistical analysis

IBM SPSS 27.0 was used to analyze the dataset. Spearman correlation analysis was conducted to assess the relationship between all demographic and clinical factors. Analysis of variance (ANOVA) was performed to compare mean scores of orthonasal and retronasal olfaction tests between people with and without parosmia, with age included as a covariate. A *p* value < 0.05 was considered statistically significant.

## Results

Among the 222 patients meeting initial inclusion criteria, 31 were excluded because they were anosmic. Another 14 were excluded because of incomplete information about qualitative olfactory disturbances, leaving 177 participants for final analyses (127 women, 50 men; mean age 45 years; see Table 1). Olfactory thresholds decreased with age (see Table 2).

**Table 1:** Demographics and chemosensory function of patients

Demographics	
Gender	127 females and 50 males
Age (years)	45.6 ± 13.6
Quantitative olfactory function^a^	
Hyposmia	127 (72%)
Normosmia	50 (28%)
Qualitative olfactory function	
Parosmia	71 (40%)
Phantosmia	78 (44%)
Gustatory function^b^	
Hypogeusia	35 (20%)
Normogeusia	133 (75%)
Data not available	9 (5%)

^a^Based on Threshold, Discrimination, Identification score

^b^Based on Taste Strip score

**Table 2:** Correlations between age and olfactory function tests in patients with and without parosmia

	Age	T	D	I	TDI	RS
Age		− 0.29*	− 0.15	− 0.06	− 0.21	0.05
T	− 0.20*		0.43**	0.11	0.78**	0.19
D	− 0.07	0.27**		0.25*	0.73**	0.42**
I	− 0.06	0.12	0.34**		0.58**	0.53**
TDI	− 0.15	0.68**	0.71**	0.68**		0.50**
RS	− 0.02	0.27**	0.35**	0.54**	0.53**	

T = Threshold, D = Discrimination, I = Identification, TDI = total score of Threshold, Discrimination and Identification. RS = Retronasal test score. Figures shown are correlation coefficients (Spearman’s r). The upper right triangle is the correlations for patients with parosmia. The lower left triangle is the correlations for patients without parosmia

*p < 0.05, **p < 0.01

**Fig. 1:**
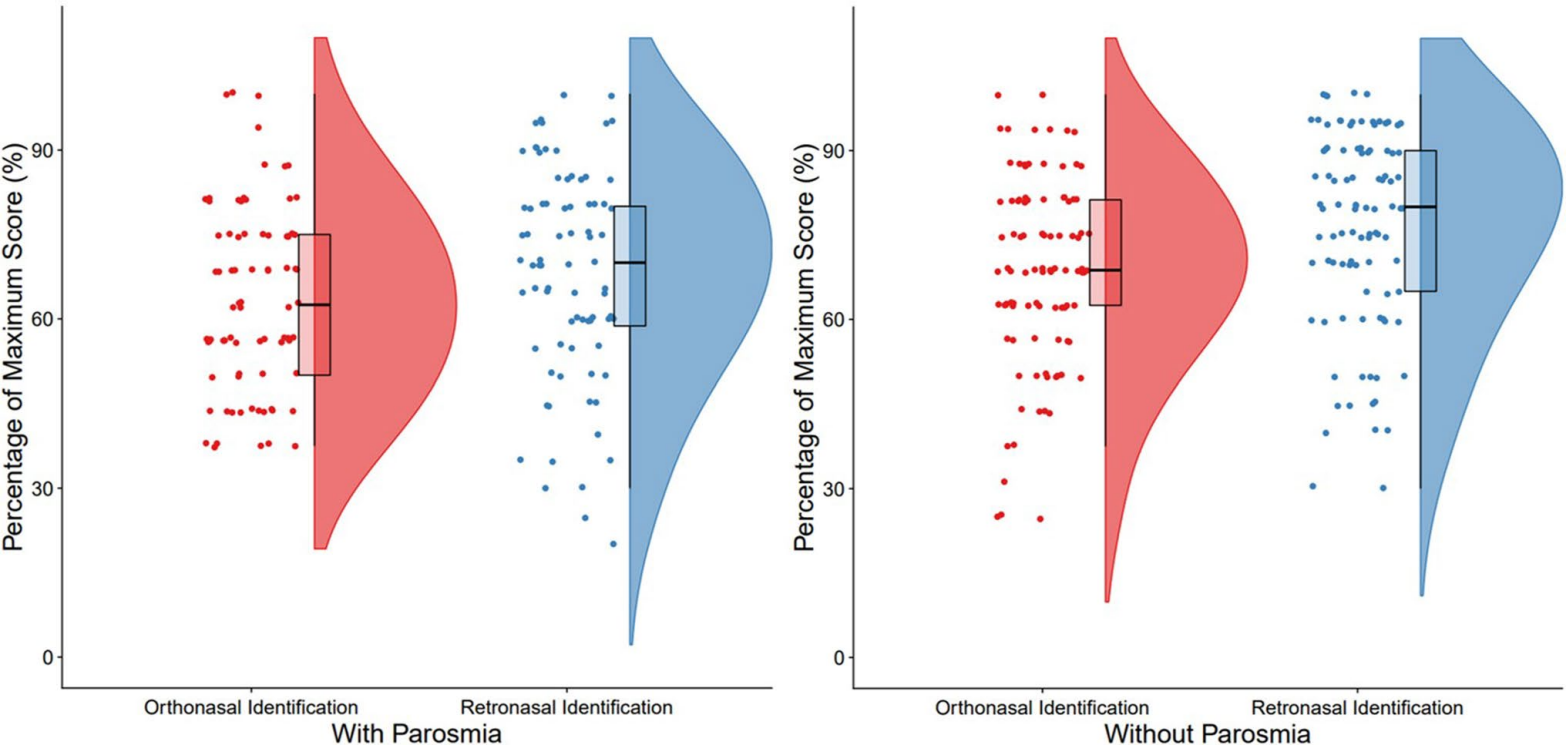
Distribution of ortho- and retronasal identification scores in patients with and without parosmia

Patients with parosmia performed significantly worse in olfactory identification tests, both ortho- and retronasally, compared to those without parosmia (orthonasal identification: *F* = 4.94, *p* = 0.03; retronasal identification: *F* = 11.95, *p* < 0.01, see Table 3). In addition, patients with parosmia had more similar scores for orthonasal and retronasal testing compared to patients with no parosmia where scores were more disparate (interaction effect between factors group [“parosmia” vs. “no parosmia”] of parosmia by identification tests [orthonasal vs. retronasal]; *F* = 4.67, *p* = 0.03, see Fig. 1).

**Table 3:** Olfactory functions comparison between patients with and without parosmia

	X ± SD (N)		*F*	*P*
	With parosmia	Without parosmia		
Threshold	5.67 ± 3.17 (71)	6.07 ± 3.10 (106)	1.31	0.25
Discrimination	10.31 ± 2.11 (71)	10.63 ± 2.40 (106)	1.12	0.29
Identification	10.06 ± 2.63 (71)	10.96 ± 2.66 (106)	4.94	0.03
TDI	26.04 ± 5.51 (71)	27.66 ± 5.70 (106)	4.52	0.04
Retronasal scores	13.35 ± 3.78 (71)	15.29 ± 3.47 (106)	11.95	< 0.01

## Discussion

Our results showed significant differences in orthonasal and retronasal odor identification scores between patients with and without parosmia. However, there was no significant difference between these groups for orthonasal threshold or discrimination scores [17–20]. Furthermore, in parosmic patients ortho- and retronasal identification sores were more similar to each other than in patients without parosmia.

Hence, it appears that parosmic patients have more trouble identifying the qualitative nature of an odor, but are relatively unimpaired in detecting or discriminating between odors. This is expected because patients with parosmia experience distortions so that they have serious difficulties identifying the nature of odors while they appear to be less handicapped perceiving odors as such. This finds its expression in similar scores between patients with and without parosmia for odor threshold and odor discrimination, tasks which do not strongly depend on the identification and naming of an odor. In contrast, the differences between the two groups come out very clearly for odor identification tasks. Accordingly, odor identification tests could be used to separate patients with and without parosmia, although the overlap is large between the two groups so that the clinical utility of such a tool would be limited. Of interest, it has previously been shown that retronasal odor identification is more difficult than orthonasal identification [21, 22]. This may help to explain the discrepancy found between patients with and without parosmia for orthonasal and retronasal tests, where the difficulties in naming odors would become more apparent in parosmics for more difficult odor identification tasks. In this sense, when contemplating the construction of a “parosmia test”, the differences between patients with and without parosmia could be highlighted by using easy and difficult odor identification tasks. In practical terms, it may be that patients with parosmia enjoy food related odors even less than odors that are orthonasally presented because they are less able to relate to retronasal than orthonasal smells.

Patients without parosmia showed relatively higher orthonasal scores than retronasal scores whereas in patients with parosmia the results from the two tests were more similar. One possible explanation for these different response patterns would relate to the distribution of the olfactory epithelium. Such unequal distributions have been shown to occur with age. It is thought that, with age, fewer olfactory receptor neurons survive in the anterior compared to the posterior part of the olfactory mucosa. This has been shown in the relatively higher success rates of biopsies from the olfactory mucosa in the posterior part [23] or the distribution of olfactory receptor neurons as shown in histological analyses from cadaver studies [24]. While these differences in epithelial distribution are clearly present, but not very pronounced, they might affect the perception of odors when presented ortho- or retronasally. For example, the perception of odors is affected by the direction of airflow reaching the mucosa due to different absorption patterns in relation to the odorants’ physicochemical properties [11, 25]. Assuming that odors, when presented retronasally, activate the olfactory system more effectively through the posterior part of the olfactory mucosa than the anterior part, such patterns could then affect the perception of odors [26]. Considering the current situation that, compared to patients without parosmia, patients with parosmia exhibit more similar scores for ortho- and retronasal odor identification, this might indicate that the viral infection affects the olfactory mucosa more in the anterior part than in the posterior part, leading to a shift in the activation of the olfactory system which in turn might facilitate the appearance of parosmic sensations. This possible unequal affection of the ortho- and retronasal mucosa could be tested in studies using biopsies or brushings from the anterior and posterior part of the olfactory mucosa. Other possibilities would be the study of ortho- or retronasal odor thresholds [27] for parosmigenic odors [28].

The present results also suggest that parosmia affects odor identification rather than odor discrimination or odor threshold. The similarity between patients with and without parosmia in terms of odor thresholds has been reported by Overbeck et al*.* [29], who investigated thresholds for 3 odorants: phenyl ethyl alcohol, a standard odor that is often used in clinical investigations, furfural mercaptan and 2,6-nonadienal, which have been shown to be relatively selective triggers of parosmia [28] and to which the sensitivity has been reported to be generally very high (e.g., Czerny, Christlbauer [30]). Interestingly, even for these odorants, there was no significant difference between patients with and without parosmia. This suggests that the generation of parosmic sensations is related to the regeneration and functioning of olfactory receptor neurons, but it does not seem to reflect a general higher sensitivity to parosmic odorants compared to hyposmic patients without parosmia.

So far, however, the origin of parosmia still remains unknown. It may result from both peripheral and central nervous changes [31–33]. For example, parosmia has been attributed to the ‘mis-wiring’ of olfactory receptor neurons to the glomeruli in the olfactory bulb, forming an incorrect or incomplete pattern during regeneration. Several mechanisms have been proposed, e.g., mistargeting of regenerating axons, changes in expression patterns of olfactory receptor neurons or incomplete regeneration patterns of olfactory receptor neurons at the level of the mucosa [34]. Other proposed mechanisms of parosmia include ephaptic firing where ‘short-circuit’ transmission occurs between neurons similar to epilepsy. Central models of parosmia have also been suggested [35], potentially involving abnormal filtering at the level of the olfactory bulb or dysfunctional central-nervous processing. The current work adds to this range of hypotheses, suggesting that a differential affect of the olfactory mucosa in the anterior–posterior axis might contribute to the distortion.

However, the present hypotheses should be interpreted with caution in populations with possible confounders, i.e. polyposis, chronic rhinosinusitis, diabetes, laryngopharyngeal reflux, or certain medications. A source of uncertainty in the present dataset is the binary response to the question regarding the presence of parosmia or phantosmia. Future studies should be performed using more detailed questionnaires.

## Conclusion

The present results may suggest that patients with parosmia are relatively more impaired when it comes to the perception of retronasal odors during eating and drinking.
